# Supplementary material for: Gain- and Loss-of-Function CFTR Alleles Are Associated with COVID-19 Clinical Outcomes
Source: Cells. 2022 Dec 16;11(24):4096. doi: 10.3390/cells11244096 (PMC9776607; doi:10.3390/cells11244096)
Supplement: Supplementary file 1 [file cells-11-04096-s001.zip › cells-2045666-supplementary.pdf]

‡ GEN-COVID Multicenter Study (https://sites.google.com/dbm.unisi.it/gen-covid)

Francesca Mari[1,2,5], Sergio Daga[1,2], Ilaria Meloni[1,2], Diana Alaverdian[1,2], Giada Beligni[1,2], Gabriella Doddato[1,2], Mirjam Lista[1,2], Debora Maffeo[1,2], Elena Pasquinelli[1,2], Loredaria Adamo[1,2], Viola Bianca Serio[1,2], Enrica Antonili[1,2], Giulia Brunelli[2], Rossella Tita[5], Maria Antonietta Mencarelli[5], Caterina Lo Rizzo[5], Anna Maria Pinto[5], Francesca Ariani[1,2,5], Francesca Montagnani[2,9], Mario Tumbarello[2,9], Ilaria Rancan[2,9], Massimiliano Fabbiani[9], Elena Bargagli[10], Laura Bergantini[10], Miriana D'Alessandro[10], Paolo Cameli[10], David Bennett[10], Federico Anedda[11], Simona Marcantonio[11], Sabino Scolletta[11], Federico Franchi[11], Maria Antonietta Mazzei[12], Susanna Guerrini[12], Edoardo Conticini[13], Luca Cantarini[13], Bruno Frediani[13], Danilo Tacconi[14], Chiara Spertilli Raffaelli[14], Arianna Emiliozzi[14], Marco Feri[15], Alice Donati[15], Raffaele Scala[16], Luca Guidelli[16], Genni Spargi[17], Marta Corridi[17], Cesira Nencioni[18], Leonardo Croci[18], Gian Piero Caldarelli[19], Davide Romani[20], Paolo Piacentini[20], Maria Bandini[20], Elena Desanctis[20], Silvia Cappelli[20], Anna Canaccini[21], Agnese Verzuri[21], Valentina Anemoli[21], Manola Pisani[21], Agostino Ognibene[22], Maria Lorubbio[22], Alessandro Pancrazzi[22], Massimo Vaghi[23], Antonella D'Arminio Monforte[24], Federica Gaia Miraglia[24], Mario U. Mondelli[25,26], Stefania Mantovani[25], Raffaele Bruno[25,26], Marco Vecchia[25], Marcello Maffezzoni[27], Enrico Martinelli[28], Massimo Girardis[29], Stefano Busani[29], Sophie Venturelli[29], Andrea Cossarizza[30], Andrea Antinori[31], Alessandra Vergori[31], Stefano Rusconi[32,33], Matteo Siano[33], Arianna Gabrieli[33], Agostino Riva[32,33], Daniela Francisci[34], Elisabetta Schiaroli[34], Carlo Pallotto[34], Saverio Giuseppe Parisi[35], Monica Basso[35], Sandro Panese[36], Stefano Baratti[36], Pier Giorgio Scotton[37], Francesca Andretta[37], Mario Giobbia[37], Renzo Scaggiante[38], Francesca Gatti[38], Francesco Castelli[39], Eugenia Quiros-Roldan[39], Melania Degli Antoni[39], Isabella Zanella[40,41], Matteo della Monica[42], Carmelo Piscopo[42], Mario Capasso[43,44], Roberta Russo[43,44], Immacolata Andolfo[43,44], Achille Iolascon[43,44], Giuseppe Fiorentino[45], Massimo Carella[46], Marco Castori[46], Giuseppe Merla[43,47], Gabriella Maria Squeo[47], Filippo Aucella[48], Pamela Raggi[49], Rita Perna[49], Matteo Bassetti[50,51], Antonio Di Biagio[50,51], Maurizio Sanguinetti[52,53], Luca Masucci[52,53], Alessandra Guarnaccia[52], Serafina Valente[54], Alex Di Florio[54], Marco Mandalà[55], Alessia Giorli[55], Lorenzo Salerni[55], Patrizia Zucchi[56], Pierpaolo Parravicini[56], Elisabetta Menatti[57], Tullio Trotta[58], Ferdinando Giannattasio[58], Gabriella Coiro[58], Fabio Lena[59], Gianluca Lacerenza[59], Cristina Mussini[60], Luisa Tavecchia[61], Lia Crotti[62,63,64,65,66], Gianfranco Parati[62,63], Roberto Menè[62,63], Maurizio Sanarico[67], Marco Gori[68,69], Francesco Raimondi[70], Alessandra Stella[70], Filippo Biscarini[71], Tiziana Bachetti[72], Maria Teresa La Rovere[73], Maurizio Bussotti[74], Serena Ludovisi[75], Katia Capitani[2,76], Simona

Dei[77], Sabrina Ravaglia[78], Annarita Giliberti[79], Giulia Gori[79], Rosangela Artuso[79], Elena Andreucci[79], Angelica Pagliazzi[79], Erika Fiorentini[79], Antonio Perrella[80], Francesco Bianchi[2,80], Paola Bergomi[81], Emanuele Catena[81], Riccardo Colombo[81], Sauro Luchi[82] Giovanna Morelli[82], Paola Petrocelli[82], Sarah Iacopini[82], Sara Modica[82], Silvia Baroni[83], Giulia Micheli[84], Marco Falcone[85], Donato Urso[85], Tommaso Matucci[85], Davide Grassi[86], Claudio Ferri[86], Franco Marinangeli[87], Francesco Brancati[88], Antonella Vincenti[89], Valentina Borgo[89], Stefania Lombardi[89], Mirco Lenzi[89], Massimo Antonio Di Pietro[90], Francesca Vichi[90], Benedetta Romanin[90], Letizia Attala[90], Cecilia Costa[90], Andrea Gabbuti[90], Alessio Bellucci[90], Marta Colaneri[91], Patrizia Casprini[92], Cristoforo Pomara[93], Massimiliano Esposito[93], Roberto Leoncini[94], Michele Cirianni[94], Lucrezia Galasso[94], Marco Antonio Bellini[95]

[1] Department of Medical Sciences, Infectious and Tropical Diseases Unit, Azienda Ospedaliera Universitaria Senese, Siena, Italy
[2] Unit of Respiratory Diseases and Lung Transplantation, Department of Internal and Specialist Medicine, University of Siena, Italy
[3] Dept of Emergency and Urgency, Medicine, Surgery and Neurosciences, Unit of Intensive Care Medicine, Siena University Hospital, Italy
[4] Department of Medical, Surgical and Neuro Sciences and Radiological Sciences, Unit of Diagnostic Imaging, University of Siena, Italy
[5] Rheumatology Unit, Department of Medicine, Surgery and Neurosciences, University of Siena, Policlinico Le Scotte, Italy
[6] Department of Specialized and Internal Medicine, Infectious Diseases Unit, San Donato Hospital Arezzo, Italy
[7] Department of Emergency, Anesthesia Unit, San Donato Hospital, Arezzo, Italy
[8] Department of Specialized and Internal Medicine, Pneumology Unit and UTIP, San Donato Hospital, Arezzo, Italy
[9] Department of Emergency, Anesthesia Unit, Misericordia Hospital, Grosseto, Italy
[10] Department of Specialized and Internal Medicine, Infectious Diseases Unit, Misericordia Hospital, Grosseto, Italy
[11] Clinical Chemical Analysis Laboratory, Misericordia Hospital, Grosseto, Italy
[12] Dipartimento di Prevenzione, Azienda USL Toscana Sud Est, Italy
[13] Dipartimento Tecnico-Scientifico Territoriale, Azienda USL Toscana Sud Est, Italy
[14] UOC Laboratorio Analisi Chimico Cliniche, Arezzo, Italy
[15] Chirurgia Vascolare, Ospedale Maggiore di Crema, Italy
[16] Department of Health Sciences, Clinic of Infectious Diseases, ASST Santi Paolo e Carlo, University of Milan, Italy

[17] Division of Clinical Immunology - Infectious Diseases, Department of Medicine, Fondazione IRCCS Policlinico San Matteo, Pavia, Italy
[18] Department of Internal Medicine and Therapeutics, University of Pavia, Italy
[19] University of Pavia, Pavia, Italy
[20] Department of Respiratory Diseases, Azienda Ospedaliera di Cremona, Cremona, Italy
[21] Department of Anesthesia and Intensive Care, University of Modena and Reggio Emilia, Modena, Italy
[22] Department of Medical and Surgical Sciences for Children and Adults, University of Modena and Reggio Emilia, Modena, Italy
[23] HIV/AIDS Department, National Institute for Infectious Diseases, IRCCS, Lazzaro Spallanzani, Rome, Italy
[24] III Infectious Diseases Unit, ASST-FBF-Sacco, Milan, Italy
[25] Department of Biomedical and Clinical Sciences Luigi Sacco, University of Milan, Milan, Italy
[26] Infectious Diseases Clinic, "Santa Maria della Misericordia" Hospital, University of Perugia, Perugia, Italy
[27] Department of Molecular Medicine, University of Padova, Italy
[28] Clinical Infectious Diseases, Mestre Hospital, Venezia, Italy.
[29] Department of Infectious Diseases, Treviso Hospital, Local Health Unit 2 Marca Trevigiana, Treviso, Italy
[30] Infectious Diseases Clinic, ULSS1, Belluno, Italy
[31] Department of Infectious and Tropical Diseases, University of Brescia and ASST Spedali Civili Hospital, Brescia, Italy
[32] Department of Molecular and Translational Medicine, University of Brescia, Italy;
[33] Clinical Chemistry Laboratory, Cytogenetics and Molecular Genetics Section, Diagnostic Department, ASST Spedali Civili di Brescia, Italy
[34] Medical Genetics and Laboratory of Medical Genetics Unit, A.O.R.N. "Antonio Cardarelli", Naples, Italy
[35] Department of Molecular Medicine and Medical Biotechnology, University of Naples Federico II, Naples, Italy
[36] CEINGE Biotecnologie Avanzate, Naples, Italy
[37] Unit of Respiratory Physiopathology, AORN dei Colli, Monaldi Hospital, Naples, Italy
[38] Division of Medical Genetics, Fondazione IRCCS Casa Sollievo della Sofferenza Hospital, San Giovanni Rotondo, Italy
[39] Laboratory of Regulatory and Functional Genomics, Fondazione IRCCS Casa Sollievo della Sofferenza
[40] Department of Medical Sciences, Fondazione IRCCS Casa Sollievo della Sofferenza Hospital, San Giovanni Rotondo, Italy
[41] Clinical Trial Office, Fondazione IRCCS Casa Sollievo della Sofferenza Hospital, San Giovanni Rotondo, Italy
[42] Department of Health Sciences, University of Genova, Genova, Italy
[43] Infectious Diseases Clinic, Policlinico San Martino Hospital, IRCCS for Cancer Research Genova, Italy

[44] Microbiology, Fondazione Policlinico Universitario Agostino Gemelli IRCCS, Catholic University of Medicine, Rome, Italy
[45] Department of Laboratory Sciences and Infectious Diseases, Fondazione Policlinico Universitario A. Gemelli IRCCS, Rome, Italy
[46] Department of Cardiovascular Diseases, University of Siena, Siena, Italy
[47] Otolaryngology Unit, University of Siena, Italy
[48] Department of Internal Medicine, ASST Valtellina e Alto Lario, Sondrio, Italy
[49] Study Coordinator Oncologia Medica e Ufficio Flussi Sondrio, Italy
[50] First Aid Department, Luigi Curto Hospital, Polla, Salerno, Italy
[51] Department of Pharmaceutical Medicine, Misericordia Hospital, Grosseto, Italy.
[52] Infectious Diseases Clinics, University of Modena and Reggio Emilia
[53] U.O.C. Medicina, ASST Nord Milano, Ospedale Bassini, Cinisello Balsamo (MI), Italy
[54] Istituto Auxologico Italiano, IRCCS, Department of Cardiovascular, Neural and Metabolic Sciences, San Luca Hospital, Milan, Italy
[55] Department of Medicine and Surgery, University of Milano-Bicocca, Milan, Italy
[56] Istituto Auxologico Italiano, IRCCS, Center for Cardiac Arrhythmias of Genetic Origin, Milan, Italy
[57] Istituto Auxologico Italiano, IRCCS, Laboratory of Cardiovascular Genetics, Milan, Italy
[58] Member of the European Reference Network for Rare, Low Prevalence and Complex Diseases of the Heart-ERN GUARD-Heart
[59] Independent Data Scientist, Milan, Italy
[60] University of Siena, DIISM-SAILAB, Siena, Italy
[61] Maasai, I3S CNRS, Université Côte d'Azur, France
[62] Laboratorio di Biologia Bio@SNS, Scuola Normale Superiore, Pisa, Italy
[63] CNR-Consiglio Nazionale delle Ricerche, Istituto di Biologia e Biotecnologia Agraria (IBBA), Milano, Italy
[64] Direzione Scientifica, Istituti Clinici Scientifici Maugeri IRCCS, Pavia, Italy
[65] Istituti Clinici Scientifici Maugeri IRCCS, Department of Cardiology, Institute of Montescano, Pavia, Italy
[66] Istituti Clinici Scientifici Maugeri IRCCS, Department of Cardiology, Institute of Milan, Italy
[67] Fondazione IRCCS Ca' Granda Ospedale Maggiore Policlinico, Milan, Italy
[68] Core Research Laboratory, ISPRO, Florence, Italy
[69] Health Management, Azienda USL Toscana Sud Est, Tuscany, Italy
[70] IRCCS C. Mondino Foundation, Pavia, Italy

[71] Medical Genetics Unit, Meyer Children's University Hospital
[72] Department of Medicine, Pneumology Unit, Misericordia Hospital, Grosseto, Italy.
[73] Department of Anesthesia and Intensive Care Unit, ASST Fatebenefratelli Sacco, Luigi Sacco Hospital, Polo Universitario, University of Milan, Milan
[74] Infectious Disease Unit, Hospital of Lucca, Italy
[75] Department of Diagnostic and Laboratory Medicine, Institute of Biochemistry and Clinical Biochemistry, Fondazione Policlinico Universitario A. Gemelli IRCCS, Catholic University of the Sacred Heart, Rome, Italy.
[76] Clinic of Infectious Diseases, Catholic University of the Sacred Heart, Rome, Italy
[77] Department of Clinical and Experimental Medicine, Infectious Diseases Unit, University of Pisa, Pisa, Italy
[78] Department of Clinical Medicine, Public Health, Life and Environment Sciences, University of L'Aquila, Italy
[79] Anesthesiology and Intensive Care, University of L'Aquila, L'Aquila, Italy
[80] Medical Genetics Unit, Department of Life, Health and Environmental Sciences, University of L'Aquila, L'Aquila, Italy
[81] Infectious Disease Unit, Hospital of Massa, Italy
[82] Infectious Diseases Unit, Santa Maria Annunziata Hospital, USL Centro, Florence, Italy
[83] Division of Infectious Diseases I, Fondazione IRCCS Policlinico San Matteo, Pavia, Italy
[84] Laboratory of Clinical Pathology and Immunoallergy, Florence-Prato, Italy
[85] Department of Medical, Surgical and Advanced Technologies "G.F. Ingrassia", University of Catania, Catania, Italy
[86] Laboratorio Patologia Clinica, Azienda Ospedaliero-Universitaria Senese
[87] Ambulatory Chronic Polipathology of Siena, Department of Medicine, Surgery and Neurosciences, University of Siena, Siena, Italy
